# Acute and Chronic Cardiopulmonary Effects of High Dose Interleukin-2 Therapy: An Observational Magnetic Resonance Imaging Study

**DOI:** 10.3390/diagnostics12061352

**Published:** 2022-05-30

**Authors:** Jakub Lagan, Josephine H. Naish, Christien Fortune, Christopher Campbell, Shien Chow, Manon Pillai, Joshua Bradley, Lenin Francis, David Clark, Anita Macnab, Gaetano Nucifora, Rebecca Dobson, Erik B. Schelbert, Matthias Schmitt, Robert Hawkins, Christopher A. Miller

**Affiliations:** 1 Wythenshawe Hospital, Manchester University NHS Foundation Trust, Wythenshawe Hospital, Southmoor Road, Wythenshawe, Manchester M23 9LT, UK; jakub.lagan@nhs.net (J.L.); christien.fortune@doctors.org.uk (C.F.); joshua.bradley@postgrad.manchester.ac.uk (J.B.); lfrancis@alliance.co.uk (L.F.); david.clark2@mft.nhs.uk (D.C.); anita.macnab@mft.nhs.uk (A.M.); gaetano.nucifora@mft.nhs.uk (G.N.); matthias.schmitt@mft.nhs.uk (M.S.); 2 Division of Cardiovascular Sciences, School of Medical Sciences, Faculty of Biology, Medicine and Health, Manchester Academic Health Science Centre, University of Manchester, Oxford Road, Manchester M13 9PL, UK; josephine.naish@manchester.ac.uk; 3 The Christie NHS Foundation Trust, Wilmslow Road, Manchester M20 4BX, UK; c.campbell9@nhs.net (C.C.); shien080@yahoo.com (S.C.); manon.pillai@christie.nhs.uk (M.P.); robert.e.hawkins@manchester.ac.uk (R.H.); 4 The Clatterbridge Cancer Centre NHS Foundation Trust, Wirral, Bebingtonm CH63 4JY, UK; 5 Liverpool Heart and Chest Hospital NHS Foundation Trust, Thomas Drive, Liverpool L14 3PE, UK; rebecca.dobson@lhch.nhs.uk; 6 Department of Medicine, University of Pittsburgh School of Medicine, Pittsburgh, PA 15213, USA; schelberteb@upmc.edu; 7 UPMC Cardiovascular Magnetic Resonance Center, Heart and Vascular Institute, Pittsburgh, PA 15213, USA; 8 Clinical and Translational Science Institute, University of Pittsburgh, Pittsburgh, PA 15260, USA; 9 Division of Cancer Sciences, Faculty of Biology, Medicine and Health, University of Manchester, Oxford Road, Manchester M13 9PL, UK; 10 Wellcome Centre for Cell-Matrix Research, Division of Cell-Matrix Biology & Regenerative Medicine, School of Biology, Faculty of Biology, Medicine & Health, Manchester Academic Health Science Centre, University of Manchester, Oxford Road, Manchester M13 9PT, UK

**Keywords:** interleukin-2, magnetic resonance imaging, myocardial fibrosis

## Abstract

High dose interleukin-2 (IL-2) is known to be associated with cardiopulmonary toxicity. The goal of this study was to evaluate the effects of high dose IL-2 therapy on cardiopulmonary structure and function. Combined cardiopulmonary magnetic resonance imaging (MRI) was performed in 7 patients in the acute period following IL-2 therapy and repeated in 4 patients in the chronic period. Comparison was made to 10 healthy volunteers. IL-2 therapy was associated with myocardial and pulmonary capillary leak, tissue oedema and cardiomyocyte injury, which resulted in acute significant left ventricular (LV) dilatation, a reduction in LV ejection fraction (EF), an increase in LV mass and a prolongation of QT interval. The acute effects occurred irrespective of symptoms. In the chronic period many of the effects resolved, but LV hypertrophy ensued, driven by focal replacement and diffuse interstitial myocardial fibrosis and increased cardiomyocyte mass. In conclusion, IL-2 therapy is ubiquitously associated with acute cardiopulmonary inflammation, irrespective of symptoms, which leads to acute LV dilatation and dysfunction, increased LV mass and QT interval prolongation. Most of these effects are reversible but IL-2 therapy is associated with chronic LV hypertrophy, driven by interstitial myocardial fibrosis and increased cardiomyocyte mass. The findings have important implications for the monitoring and long term impact of newer immunotherapies. Future studies are needed to improve risk stratification and develop cardiopulmonary-protective strategies.

## 1. Introduction

Despite the potential for cardiopulmonary toxicity, there is a paucity of prospective systematic in vivo data regarding the effect that high dose interleukin-2 (IL-2) therapy has on cardiopulmonary structure and function in humans.

Renal cell cancer (RCC) is the seventh most common cancer worldwide [1,2]. The incidence, which is highest in North America and Western Europe, is increasing, indeed the number of new cases increased by almost 50% in the UK over the last decade [1,2]. Nearly 20% of patients have distant metastases at the time of diagnosis and 20–40% relapse after nephrectomy [3].

High dose IL-2 therapy was one of the earliest oncologic immunotherapies and it is an effective treatment of metastatic RCC, associated with an objective response in tumour size in over 40% of selected patients and with durable complete remissions in around half of those patients [4,5]. However, it can be associated with significant cardiopulmonary side effects, including hypotension, arrhythmia, myocardial ischaemia and infarction, cardiomyopathy, pleural effusions and respiratory distress syndrome [6,7,8,9,10]. IL2-induced myocarditis, a relatively common reason for discontinuing IL-2 therapy, is clinically evident in up to approximately 25% of patients [4,7,11,12], however autopsy data suggest it may occur more frequently and can lead onto chronic myocardial fibrosis [13,14].

This study aimed to evaluate the acute and chronic effects of high dose IL-2 therapy on cardiopulmonary structure and function. Evaluation included novel combined cardiopulmonary magnetic resonance imaging (MRI), which provides unique in vivo insight into human cardiac and pulmonary structure, function and tissue characteristics.

## 2. Materials and Methods

### 2.1. Study Design

This prospective research study aimed to investigate the acute and chronic cardiopulmonary effects of IL-2 therapy in patients receiving IL-2 therapy for metastatic RCC. Findings were compared to those in prospectively recruited matched healthy volunteers.

### 2.2. Study Population

Consecutive consenting patients undergoing IL-2 therapy for metastatic RCC at The Christie NHS Foundation Trust, UK were prospectively recruited. Patients were planned to receive 0.6 million IL-2 units/kg every 8 h for 5 days (“week one”), followed by a 10 day break, and then a further 0.6 million IL-2 units/kg every 8 h for 5 days (“week two”). All patients had normal left ventricular (LV) function and no evidence of inducible ischaemia on dobutamine stress echocardiography before starting IL-2 therapy.

Age- and gender-matched healthy volunteers with no cardiovascular symptoms, no history of medical conditions and normal electrocardiogram (ECG) were prospectively recruited to provide control data.

Exclusion criteria included: contraindication to MRI (including claustrophobia), hypersensitivity to gadolinium-based contrast agent (GBCA) and estimated glomerular filtration rate (eGFR) less than 40 mL/min/1.73 m^2^.

### 2.3. Study Procedures

Participants underwent ECG, blood sampling for blood count, renal function, c-reactive protein and high sensitivity troponin I and cardiopulmonary MRI. Blood sampling was performed in the 60 min period before the cardiopulmonary MRI. Patients receiving IL-2 therapy underwent the investigations on two occasions: 1. Within 5 days of receiving IL-2 therapy (“acute”); 2. A minimum of 7 weeks after receiving the final dose of IL-2 therapy (“chronic”). Healthy volunteers were scanned once.

### 2.4. Cardiopulmonary MRI

MRI was performed at 1.5T scanner (Avanto, Siemens Medical Imaging, Hartford, CT, USA). Scan duration was 60 min. The MRI protocol is described in the Supplementary Materials. It was also previously described in detail [15,16]. In brief, it included: myocardial steady-state free precession cine imaging, myocardial and pulmonary native T1 mapping, myocardial T2 mapping, myocardial and pulmonary dynamic contrast enhanced MRI (DCE-MRI), post contrast myocardial T1 mapping and LGE imaging.

### 2.5. MRI Analysis

MRI analysis is described in the Supplementary Materials and was also described previously in detail (15, 16). In brief, cardiac volumetric analysis was performed using Circle CVI42 (Circle Cardiovascular Imaging, Calgary, AB, Canada) according to guidelines [17]. Left and right atrial areas were measured in four chamber cine view at end systole. Parametric maps were analysed in Horos (Horos2K v2.2.0 The Horos Project). Myocardial extracellular volume fraction (ECV) was calculated using same-day haematocrit as described previously [18]. LV extracellular matrix mass (g) was calculated by multiplying LV mass by ECV [19]. LV cellular mass (g) was calculated by multiplying LV mass by (100%-ECV). DCE imaging was analysed using a custom written Matlab code (v9.0, The MathWorks, Natick, MA, USA). Contrast agent kinetics were modelled using an extended version of a Kety model to calculate myocardial and pulmonary capillary permeability (K^trans^) and pulmonary extracellular volume fraction (Ve) [20]. Pulmonary blood flow (F) was calculated by deconvolution of the first pass dynamic data as described previously [20,21].

### 2.6. Statistical Analysis

There were no data upon which to base a power calculation in this population. Using data from other populations, 10 patients were required to detect an absolute minimum difference, between acute and chronic scans, of 2% in terms of absolute change in myocardial ECV, with 80% power at a 5% significance level (2-sided), assuming a standard deviation of the within-patient differences equal to 2% [18,22]. Data distribution was determined using the Shapiro-Wilk test. For normally distributed variables, data are summarised using mean +/− standard deviation and compared using parametric *t*-tests. For variables not distributed normally, data are summarized using median values and interquartile ranges (IQR) and compared using non-parametric tests. Correlation analysis was performed using Pearson or Spearman correlation as appropriate. A subgroup analysis including only asymptomatic patients was pre-specified. Analyses were performed using SPSS (version 22, IBM, Cambridge, MA, USA).

## 3. Results

### 3.1. Participant Characteristics

Seven patients undergoing IL-2 therapy were recruited (Table 1). All patients were male; median age 58 (interquartile range 56–60) years. Five patients were included following “week one” of IL-2 therapy and two were included following “week two”. Patients received a median of 10 (6–11) doses of 0.6 million IL-2 units/kg in the week prior to inclusion. Two patients developed cardiopulmonary symptoms during IL-2 therapy: one developed new onset atrial fibrillation that was associated with palpitations and one experienced chest pain. The other 5 patients did not develop any cardiopulmonary symptoms.

### 3.2. Acute Cardiopulmonary Effects of IL-2 Therapy

Mean time between the final dose of IL-2 and the acute evaluation was 2.7 ± 1.5 days. Patients receiving IL-2 demonstrated significantly higher circulating inflammatory markers and longer corrected QT interval (430 ms vs 397 ms; *p* = 0.001; Table 2) compared to healthy volunteers. High sensitivity troponin I was elevated in 4 patients (57%).

Acute IL-2 therapy was associated with significant LV dilatation, a significant reduction in LV ejection fraction (EF) (56% vs. 64%; *p* = 0.013) and significantly increased LV mass (51 ± 7 g/m^2^ vs. 67 ± 9 g/m^2^; *p* = 0.001) in comparison to healthy volunteers. There were no differences in RV indices.

**Figure 1:**
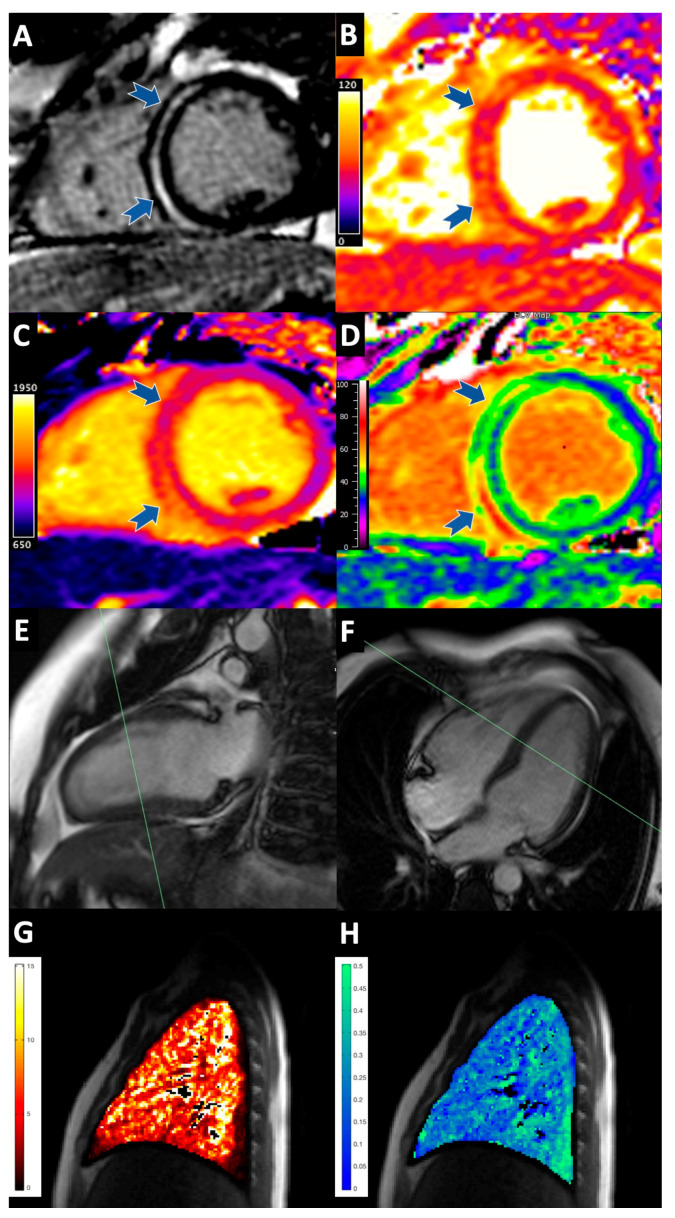
Acute cardiopulmonary tissue manifestations of high dose IL-2 therapy. (**A**–**F**) Myocardium; (**G**–**H**) Right lung. (**A**) Prominent mid-wall septal non-ischaemic enhancement is seen on late gadolinium enhancement, diastolic phase (blue arrows); (**B**) Elevated myocardial T2 (septum 64 ms; lateral wall 47 ms) and (**C**) T1 (septum 1353 ms, lateral wall 1039 ms) are seen on diastolic T2 and T1 maps respectively. (**D**) Markedly expanded myocardial extracellular volume (septum 66%, lateral wall 28%) is seen on diastolic extracellular volume mapping. (**E**,**F**) End diastolic long axis cine imaging showing the position of LV short axis slices. (**G**) Increased pulmonary tissue blood flow (5.8 mL blood/mL tissue/min) and (**H**) expanded pulmonary extracellular volume (Ve; 25%) are seen on perfusion and extracellular volume maps respectively.

Acute IL-2 therapy was associated with significantly higher native myocardial T1 (1086 ms vs. 1000 ms; *p* < 0.001), T2 (54 ms vs. 49 ms; *p* < 0.001), ECV (29.3% vs. 24.3%; *p* = 0.001) and a non-significant increase in K^trans^ (Table 2, Figure 1). All patients receiving IL-2 therapy exhibited non-ischaemic LGE, the distribution of which was mid wall, epicardial and/or RV insertion point. No patient had evidence of acute myocardial infarction on LGE imaging.

There was a strong positive correlation between circulating high sensitivity troponin I level and myocardial ECV (r = 0.825, *p* = 0.022), T1 (r = 0.935, *p* = 0.002) and amount of non-ischaemic LGE (r = 0.898, *p* = 0.006).

Acute IL-2 therapy was associated with significantly higher pulmonary tissue perfusion (4.07 vs. 2.32mL blood/mL tissue/min; *p* = 0.048) and Ve (30 vs. 19%; *p* = 0.035), and strong trends towards higher pulmonary T1 and K^trans^.

In a subgroup analysis confined to those patients who did not develop cardiopulmonary symptoms with acute IL-2 therapy (5 patients), corrected QT interval was significantly longer than in healthy volunteers and two patients demonstrated elevated high sensitivity troponin I levels (Table 3). IL-2 therapy was associated with significant LV dilatation, a strong trend towards a reduction in LV EF and significantly increased LV mass in comparison to healthy volunteers. IL-2 therapy was also associated with significantly higher native myocardial T1, T2 and ECV, and all patients exhibited non-ischaemic LGE. Pulmonary K^trans^ was significantly elevated and pulmonary Ve and tissue perfusion showed strong trends towards being elevated. 

### 3.3. Chronic Cardiopulmonary Effects of IL-2 Therapy

Four participants underwent repeat assessment in the chronic period post-IL-2 therapy. One participant unfortunately passed away due to cancer progression and two withdrew their consent for the follow up MRI scans. Mean time between the final dose of IL-2 and the chronic evaluation was 91 ± 25 days. At the time of the chronic evaluation, high sensitivity troponin I had returned to normal in all patients and QT interval prolongation had resolved (Table 2). White cell count had also returned to normal although C reactive protein remained mildly elevated.

**Figure 2:**
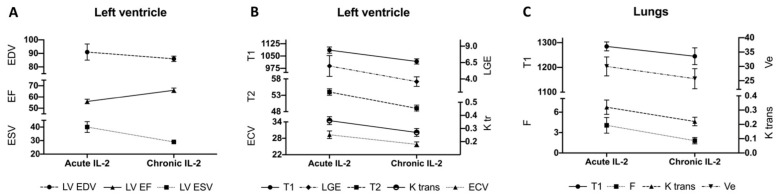
Chronic effects of IL-2 therapy on cardiopulmonary structure, function and tissue character. (**A**) In the chronic period post-IL2 therapy, left ventricular end diastolic volume (EDV; mL/m^2^) and end systolic volume (ESV; mL/m^2^) reduced, leading to an improvement (normalisation) in LV ejection fraction (EF; %). (**B**) Myocardial T1 (ms) and T2 (ms), which reflect myocardial oedema, and capillary permeability (Ktrans; min-1), also returned to normal in the chronic period. Myocardial extracellular volume (ECV; %) and late gadolinium enhancement mass (LGE; g) both improved compared to the acute phase, but remained elevated, in keeping with diffuse and focal myocardial fibrosis respectively. (**C**) Pulmonary blood flow (F; ml blood/mL tissue/min), measurement of pulmonary tissue oedema (T1 (ms) and extracellular volume fraction (Ve, %)) and capillary permeability (Ktrans; min-1) all returned to normal in the chronic period post-IL2 therapy.

In the chronic period post-IL2 therapy, LV EF improved significantly (acute IL-2: 56% vs. chronic IL-2: 66%, *p* = 0.02), returning back to normal. This was predominantly driven by an improvement in LV end systolic volume (Figure 2).

LV mass remained significantly elevated in the chronic period post-IL-2 therapy (chronic IL-2: 65 ± 9 g/m^2^ vs. control: 51 ± 7 g/m^2^; *p* = 0.008). This was driven in particular by increased extracellular matrix mass (chronic IL-2: 38 ± 8 g vs. control: 26 ± 5 g; *p* = 0.005) as well as increased cardiomyocyte mass (chronic IL-2: 104 ± 13 g vs. control: 82 ± 16 g; *p* = 0.027). All patients continued to exhibit non-ischaemic LGE. Myocardial T1 and T2 and all pulmonary indices returned to normal (Figure 2).

## 4. Discussion

In the first systematic evaluation of the acute and chronic cardiopulmonary effects of high dose IL-2 therapy, this study found that high dose IL-2 therapy is associated with a significant acute inflammatory cardiopulmonary injury, which leads onto chronic cardiac maladaptation. High dose IL-2 was associated with acute cardiopulmonary capillary leak, myocardial and pulmonary tissue oedema and cardiomyocyte injury, which resulted in an acute increase in LV mass, LV dilatation, reduced LV function and QT prolongation. The acute injury occurred irrespective of symptoms. In the chronic phase post-IL2 therapy, LV size, ejection fraction and ECG measurements returned to normal, but LV hypertrophy ensued, driven by a combination of focal replacement and diffuse interstitial myocardial fibrosis and increased cardiomyocyte mass. The findings are novel and provide the basis for further investigation aimed at stratifying the risk of developing cardiopulmonary complications and evaluating cardiopulmonary-protective strategies. The findings also have important implications for the monitoring and long term impact of newer immunotherapies.

The mechanism of action of IL-2 is to promote the generation of lymphokine-activated killer (LAK) cells, which are cytotoxic for tumour cells [23]. LAK cells are also responsible for many of the side effects of IL-2 therapy. Capillary leak syndrome, which is seen in more than 50% of patients receiving IL-2 therapy, occurs both directly as a result of interaction with endothelial cells via CD25 [24] and indirectly via LAK cell-stimulated production of vasoactive lymphokines, which increase capillary permeability and decrease vascular tone [23,25,26].

The proposed mechanism of IL-2-related myocardial injury includes the directly mediated increase in vascular permeability and the migration of LAK cells into the myocardium with subsequent release of proinflammatory cytokines and chemo-attraction of other inflammatory cells. This results in activation of the myocardial capillary endothelium and expression of adhesion molecules with subsequent disruption of the endothelium, capillary leak and lymphocytic plugging of microvessels. LAK cells also have a direct cytotoxic effect on cardiomyocytes [27]. Autopsy studies have shown that acute myocardial inflammation is common in the first 5 days following IL-2 therapy, being seen in up to 75% of patients regardless of symptoms or cause of death [14]. Autopsy data also demonstrate that approximately 50% of patients go on to develop myocardial fibrosis following IL-2 therapy, and that the fibrotic process begins early post-therapy [13,14]. The reported incidence of clinically evident IL-2 related myocarditis varies considerably (0.6–25%), but is substantially lower than that reported at autopsy [4,7,11,12].

Previous human in vivo data regarding the cardiopulmonary effect of high dose IL-2 therapy are largely limited to case reports. One small study found the myocardial injury following IL-2 therapy was similar to that of infectious myocarditis [28]. IL-2 therapy has previously been reported to have a variable effect on LV function [8,29,30,31].

The multiparametric nature of MRI, i.e., its ability to measure multiple aspects of tissue structure, function, injury and adaptation within one scan, without radiation, means that it is uniquely placed to elucidate underlying disease mechanisms and understand their relationships. With the advent of parametric mapping and ECV assessment, MRI sensitivity and specificity in detecting myocardial pathologies has been greatly increased [19]. In the presence of myocardial inflammation and increased myocardial free water content (i.e., oedema), high T1, T2 and ECV values are expected. In the presence of interstitial myocardial fibrosis, T1 and ECV values are high while T2 values are normal.

The findings of the current study are in keeping with pathophysiology and autopsy findings, whilst also providing new insight. Myocardial inflammation was ubiquitous in the acute period following IL-2 therapy, manifesting as capillary leak (elevated K^trans^), myocardial oedema (elevated T1, T2 and ECV) and cardiomyocyte injury (elevated high sensitivity troponin). The higher prevalence of myocardial inflammation in the current study compared to the autopsy data is likely because MRI provides greater heart coverage (less sampling error) and assesses multiple aspects of inflammation.

The acute myocardial injury occurred independently of symptoms, which highlights the unreliability of symptoms for diagnosing cardiopulmonary complications and demonstrates that substantial myocardial injury can occur in their absence. While there were strong relationships between cardiomyocyte injury (high sensitivity troponin level) and myocardial oedema (T1 and ECV), myocardial oedema was also evident in patients with normal high sensitivity troponin levels demonstrating that it can occur independently of cardiomyocyte necrosis. 

The acute myocardial injury had a significant impact on LV structure and function, specifically LV dilatation, reduced LV function and increased LV mass. In four (57%) patients, LV EF dropped below the widely used 55% normality threshold, although no patient developed more than mild LV dysfunction. Symptomatic patients did develop numerically greater LV dysfunction (acute LV EF symptomatic patients: 51 ± 2% vs. asymptomatic patients 58 ± 6%) but the small number of patients with symptoms (2) precludes meaningful statistical comparison. The finding that IL-2 therapy acutely induces relative QT interval prolongation may be a potential mechanism for the arrhythmias that have previously been reported following IL-2 therapy.

The current study demonstrates that at approximately 3 months following IL-2 therapy, the acute myocardial inflammation has resolved and LV EF and QT interval have normalised. However, the study shows, for the first time, that IL-2 therapy is associated with subsequent LV hypertrophy. This is driven by focal replacement and diffuse interstitial myocardial fibrosis, consistent with the previous autopsy data, and increased cardiomyocyte mass. Myocardial fibrosis is strongly associated with adverse outcome across a range of cardiovascular conditions [32,33] and further work is required to determine the association between myocardial fibrosis and outcome in patients receiving IL-2 therapy.

No patient developed a myocardial infarction following IL-2 therapy in this study. In early studies, 1–6% of patients suffered a myocardial infarction following IL-2 therapy [6,7,34], but the incidence has since declined as a result of pre-therapy functional cardiac imaging, as was performed in all patients in the current study [4].

The lungs are commonly affected by capillary leak syndrome and the pulmonary findings demonstrated here, including increased pulmonary tissue blood flow, capillary permeability and oedema, are consistent with capillary leak syndrome [23]. All pulmonary measurements returned to normal in the chronic period, in keeping with its reversible nature.

The clinical implications of this study require further discussion and investigation. The clinical approach to IL-2 related myocarditis is mainly supportive and includes termination of IL-2 therapy [35]. In cases of LV dysfunction, usual heart failure therapy is often utilized [36]. Although immunosuppressive treatment is being used in immune checkpoint inhibitors-related myocarditis [37], there is currently no evidence that it is effective in patients on IL-2 treatment. Clinically evident myocarditis following IL-2 therapy is also considered a contraindication to future therapy. The current study shows that myocarditis is ubiquitous following IL-2 therapy, and that symptoms are a poor guide of the severity of the myocardial injury. However, no patient developed more than mildly reduced LV EF, and it returned to normal by 3 months in all cases. Identification of those patients who are at risk of IL-2 related cardiotoxicity has also been difficult and further investigation is required to identify factors that predispose to cardiopulmonary side effects in order to improve risk stratification, and to evaluate cardiopulmonary protective strategies. Screening functional cardiac imaging remains a widely adapted approach to rule out pre-existing significant coronary artery disease [38] and has been effective in reducing the rate of myocardial infarction following IL-2 therapy; a similar strategy to identify those patients who are at risk of developing the most significant inflammatory LV dysfunction would be highly beneficial. Currently, there are no established biomarkers that would be helpful in predicting the risk of IL-2 related myocarditis.

The findings also have important implications for newer immunotherapies. Immune checkpoint inhibitors are known to be associated with cardiovascular complications. Myocarditis is considered the most lethal form of immune-related adverse event with fatality rate of 27–46% [39]. Other cardiovascular complications of immune checkpoint inhibitors reported in the literature include Takotsubo cardiomyopathy, myocardial ischemia and infarction and pericardial disease [39]. Registry data suggest the incidence of myocarditis is low but the diagnosis of myocarditis in such studies is often dependent on symptoms, ECG changes and troponin elevation whereas the current study demonstrates that significant myocarditis can occur in their absence, thus the true incidence may be higher [40]. Troponin and echocardiography have been suggested as methods to monitor for cardiac toxicity, but, as demonstrated, troponin is neither sensitive or specific for myocarditis and echocardiography is insensitive to small changes in LV EF and not able to detect the myocardial injury shown here. MRI screening should be considered. Furthermore, given the curative potential of checkpoint inhibitors, the long-term impact of myocardial structural derangements in survivors requires investigation. Similarly, cardiac MRI studies could be helpful to inform further development of novel IL-2 variants, such as mutated forms of Interleukin-2, antibody-interleukin-2 fusions and antibody-interleukin-2 complexes.

Finally, the study serves as a disease model of myocardial injury, demonstrating that diffuse myocardial injury leads to permanent structural derangement, despite the ostensible functional recovery. Thus, recovery is indeed incomplete following the initial insult, and the risk of the associated adverse cardiovascular outcome likely persists. The metrics employed here may better inform myocardial injury following other potentially toxic exposures.

## 5. Conclusions

High dose IL-2 therapy is ubiquitously associated with acute cardiopulmonary inflammation, irrespective of symptoms, which results in acute LV dilatation and dysfunction, increased LV mass and QT interval prolongation. The cardiopulmonary inflammation, LV dysfunction and QT interval prolongation are all reversible, but IL-2 therapy is associated with subsequent chronic LV hypertrophy, driven by focal replacement and diffuse interstitial myocardial fibrosis and increased cardiomyocyte mass. The study has important implications for the monitoring and long term impact of newer immunotherapies, and provides the basis for further investigation aimed at improving risk stratification and evaluating cardiopulmonary-protective strategies. 

## 6. Limitations

The number of patients included was small. New therapies for metastatic RCC were introduced during the study which led to reduced numbers of patients undergoing IL-2 therapy [41,42]. Nevertheless, significant results were demonstrable. It was not possible to perform MRI scanning before IL-2 therapy was initiated and study participants could not act as their own controls. It was also not possible to obtain histological data as it was not felt ethically acceptable to conduct lung and endomyocardial biopsies as part of this study. Although normal reported values for the used parametric sequences on 1.5 T Siemens scanner are available in literature (native T1: 885–1059 ms, ECV: 20–32%, native T2: 45–65 ms [17]), they can differ between scanner set-ups. Therefore these literature-derived values were not used for comparison in this study. Instead patients were compared to matched healthy volunteers.

## Figures and Tables

**Table 1:** Participant characteristics.

Parameter	Control (n = 10)	Acute IL-2 (n = 7)	*p*-Value *
**Demographics**			
Age (Years)	51 (44–58)	58 (56–60)	0.172
Gender (Males, %)	10 (100%)	7 (100%)	1.000
BSA (m^2^)	2.04 (1.98–2.25)	2.09 (2.06–2.34)	0.283
**RCC characteristics**			
Pulmonary metastases	0 (0%)	7 (100%)	
Lymph nodes metastases	0 (0%)	4 (57%)	
Previous nephrectomy	0 (0%)	7 (100%)	
**Comorbidities**			
Paroxysmal atrial fibrillation	0 (0%)	1 (14%)	
Previous pulmonary embolism	0 (0%)	1 (14%)	
Multiple sclerosis	0 (0%)	1 (14%)	
Asthma	0 (0%)	1 (14%)	
Hypertension	0 (0%)	1 (14%)	
Current smoker	0 (0%)	1 (14%)	
Dyslipidaemia	0 (0%)	1 (14%)	

Results presented as mean ± standard deviation or median (interquartile range) depending on data distribution. * Acute IL-2 patients vs healthy volunteers (controls)**.** BSA: body surface area; IL-2: interleukin-2; RCC: Renal cell carcinoma.

**Table 2:** Acute and chronic effects of IL-2 therapy.

Parameter	Controls (n = 10)	Acute IL-2 (n = 7)	*p*-Value *	Chronic IL-2 (n = 4)	*p*-Value ^†^	*p*-Value ^∆^
**Laboratory findings**						
CRP (mg/L)	2 (0–2)	17 (13–40)	0.001	9 (7–10)	0.019	0.068
WBC (×10^9^/L)	6 (6–7)	29 (11–92)	0.001	2 (0–4)	1.000	0.068
hsTnI (ng/L)	4 (4–4)	37 (4–610)	0.017	6 (3–14)	1.000	0.066
Potassium (mmol/L)	4.0 ± 0.2	4.0 ± 0.4	0.951	4.1 ± 0.2	0.408	0.929
Urea (mmol/L)	5.8 ± 0.9	6.5 ± 2.4	0.458	7.3 ± 1.0	0.022	0.943
Creatinine (µmol/L)	82 (79–88)	115 (105–117)	0.015	131 (111–141)	0.005	0.109
eGFR (mL/min/1.73m^2^)	86 (81–91)	56 (55–63)	0.009	49 (45–60)	0.004	0.083
**ECG findings**						
PR duration (ms)	160 (140–160)	160 (160–180)	0.392	170 (145–195)	0.274	0.564
QRS duration (ms)	80 (80–100)	100 (80–100)	0.681	100 (85–100)	0.327	0.317
QTc (ms)	397 ± 12	430 ± 19	0.001	407 ± 15	0.203	0.047
**Left ventricle**						
LV EDV/BSA (mL/m^2^)	78 ± 13	91 ± 16	0.076	86 ± 4	0.288	0.221
LV ESV/BSA (mL/m^2^)	28 ± 7	40 ± 9	0.010	29 ± 3	0.904	0.073
LV EF (%)	64 ± 5	56 ± 6	0.013	66 ± 3	0.390	0.02
LV mass/BSA (g/m^2^)	51 ± 7	67 ± 9	0.001	65 ± 9	0.008	0.123
**Right ventricle**						
RV EDV/BSA (mL/m^2^)	86 ± 14	91 ± 17	0.545	96 ± 9	0.249	0.921
RV ESV/BSA (mL/m^2^)	37 ± 8	39 ± 12	0.554	41 ± 8	0.324	0.704
RV EF (%)	58 ± 5	57 ± 7	0.855	57 ± 5	0.820	0.216
**Atria**						
LA area/BSA (cm^2^/m^2^)	12 ± 1	12 ± 2	0.847	13 ± 1	0.479	0.882
RA area/BSA (cm^2^/m^2^)	12 ± 2	11 ± 2	0.550	11 ± 2	0.519	0.828
**Tissue characterisation**						
Cardiac indices						
LGE (g)	0.00 (0.00–0.00)	5.98 (2.02–6.57)	<0.001	3.60 (1.45–3.92)	<0.001	0.068
T2 (ms)	49 ± 2	54 ± 2	<0.001	49 ± 2	0.814	0.007
T1 (ms)	1000 ± 23	1086 ± 50	<0.001	1017 ± 32	0.312	0.011
ECV (%)	24.3 (22.6–25.6)	29.3 (28.9–33.6)	0.001	25.8 (25.3–28.4)	0.066	0.068
Extracellular matrix mass (g)	26 ± 5	46 ± 6	<0.001	38 ± 8	0.005	0.071
Cellular mass (g)	82 ± 16	101 ± 18	0.035	104 ± 13	0.027	0.309
K^trans^ (min^−1^)	0.29 ± 0.08 ^∂^	0.36 ± 0.07	0.103	0.27 ± 0.05	0.675	0.083
Pulmonary indices						
T1 (ms)	1237 ± 35 ^∂^	1285 ± 49	0.056	1245 ± 67	0.827	0.249
Ve (%)	19.0 (15.6–26.6) ^∂^	30.0 (25.9–34.3)	0.035	25.7 (19.7–32.7)	0.131	0.068
K trans (min^−1^)	0.22 ± 0.06 ^∂^	0.32 ± 0.14	0.089	0.22 ± 0.07	0.957	0.104
F (mL blood/mL tissue/min)	2.32 (1.76–3.10) ^∂^	4.07 (2.67–4.94)	0.048	1.81 (1.12–2.83)	0.345	0.068

Results presented as mean ± standard deviation or median (interquartile range) depending on data distribution. * Acute IL-2 patients vs. healthy volunteers (controls); ^†^ Chronic IL-2 patients vs controls; ^∆^ Acute vs Chronic IL-2 patients. ^∂^ n = 7. CRP: C reactive protein; ECG: electrocardiogram; ECV: cardiac extracellular volume fraction; EDV: end systolic volume; EF: ejection fraction; eGFR: estimated glomerular filtration rate; ESV: end systolic volume; F: pulmonary tissue blood flow; hsTnI: high sensitivity troponin I; Ktrans: transfer constant; LA: left atrium; LGE: late gadolinium enhancement; LV: left ventricle; QTc: corrected QT interval; RA: right atrium; RV: right ventricle; Ve: pulmonary extracellular volume fraction; WBC: white blood cell. Other abbreviations as per Table 1.

**Table 3:** Acute effects of IL-2 therapy in asymptomatic patients.

Parameter	Controls (n = 10)	Asyptomatic IL2 (n = 5)	*p*-Value *
**Laboratory findings**			
CRP (mg/L)	6 (6–7)	21 (16–48)	0.003
WBC (×10^9^/L)	2 (0–2)	29 (12–108)	0.002
TnI (ng/L)	4 (4–4)	4 (4–409)	0.063
**ECG findings**			
PR duration (ms)	160 (140–160)	160 (160–170)	0.315
QRS duration (ms)	80 (80–100)	100 (80–100)	0.591
QTc (ms)	397 ± 12	440 ± 10	<0.001
**LV**			
LV EDV/BSA (mL/m^2^)	78 ± 13	95 ± 17	0.052
LV ESV/BSA (mL/m^2^)	28 ± 7	40 ± 11	0.027
LV EF (%)	64 ± 5	58 ± 6	0.078
LV mass/BSA (g/m^2^)	51 ± 7	66 ± 11	0.006
**RV**			
RV EDV/BSA (mL/m^2^)	86 ± 14	95 ± 18	0.335
RV ESV/BSA (mL/m^2^)	37 ± 8	40 ± 12	0.539
RV EF (%)	58 ± 5	59 ± 6	0.754
**Atria**			
LA area/BSA (cm^2^/m^2^)	12 ± 1	13 ± 2	0.305
RA area/BSA (cm^2^/m^2^)	12 ± 2	12 ± 2	0.905
**Tissue characterisation**			
Cardiac indices			
LGE (g)	0.00 (0.00–0.00)	5.53 (1.94–6.28)	<0.001
T2 (ms)	49 ± 2	54 ± 2	0.002
T1 (ms)	1000 ± 23	1063 ± 20	<0.001
ECV (%)	24.3 (22.6–25.6)	29.3 (28.9–33.1)	0.002
K^trans^ (min^−1^)	0.29 ± 0.08	0.38 ± 0.07	0.07
Extracellular matrix mass (g)	26 ± 5	44 ± 6	<0.001
Cellular mass (g)	82 ± 16	99 ± 17	0.066
Pulmonary indices			
T1 (ms)	1237 ± 35	1273 ± 51	0.176
Ve (%)	19.0 (15.6–26.6)	31.5 (21.5–37.9)	0.062
K^trans^ (min^−1^)	0.22 ± 0.06	0.36 ± 0.14	0.037
F (mL blood/mL tissue/min)	2.32 (1.76–3.10)	4.15 (2.62–8.05)	0.062

Results presented as mean ± standard deviation or median (interquartile range) depending on data distribution. * Asymptomatic IL-2 patients vs healthy volunteers (controls). Abbreviations as per Table 1 and Table 2.

## Data Availability

The datasets generated and/or analysed during the current study are available from the corresponding author on reasonable request.

## Supplementary Materials

The following supporting information can be downloaded at: https://www.mdpi.com/article/10.3390/diagnostics12061352/s1. Supplementary Materials File S1: MRI acquisition protocol and MRI analysis. References [43,44,45,46] was cited in supplementary materials.
