# Supplementary material for: Acute and Chronic Cardiopulmonary Effects of High Dose Interleukin-2 Therapy: An Observational Magnetic Resonance Imaging Study
Source: Diagnostics (Basel). 2022 May 30;12(6):1352. doi: 10.3390/diagnostics12061352 (PMC9221588; doi:10.3390/diagnostics12061352)
Supplement: Supplementary file 1 [file diagnostics-12-01352-s001.zip › diagnostics-1715415-supplementary.pdf]

Acute and chronic cardiopulmonary effects of high dose interleukin-2 therapy: an observational magnetic resonance imaging study

Supplementary material

1. Cardiopulmonary MRI acquisition protocol

MRI was performed at 1.5T (Avanto, Siemens Medical Imaging). The scan duration was 60 minutes. The protocol was previously described in detail (Lagan J, Naish JH, Bradley J, Fortune C, Palmer C, Clark D, et al. Cardiac involvement in cystic fibrosis evaluated using cardiopulmonary magnetic resonance. Int J Cardiovasc Imaging. 2022:1-11 [Online ahead of print], Lagan J, Naish JH, Fortune C, Bradley J, Clark D, Niven R, et al. Myocardial involvement in eosinophilic granulomatosis with polyangiitis evaluated with cardiopulmonary magnetic resonance. Int J Cardiovasc Imaging. 2021;37(4):1371-81.). and it included the following sequences:

1. Steady-state free precession (SSFP) cine imaging of the heart (standard long- and short-axis views) to provide measurement of cardiac function.
2. T1 mapping (by MOdified Look-Locker Inversion Recovery (MOLLI)) at basal and mid left ventricular (LV) short axis level before and 15 minutes following the final bolus of GBCA (see below) to provide evaluation of myocardial oedema and fibrosis. T2 mapping (by T2-prepared SFFP) at the same positions to provide further assessment of myocardial oedema. Late gadolinium enhancement (LGE) imaging beginning at 6 minutes following the final GBCA bolus (see below) to assess for focal replacement myocardial fibrosis.
3. T1 mapping (by MOLLI) in the central sagittal plane of both lungs to provide evaluation of lung tissue injury.
4. Dynamic contrast enhanced (DCE) imaging using free-breathing 2D saturation-recovery fast low angle shot (FLASH) dynamic acquisitions in four planes (basal and mid LV short axis, left and right lung central sagittal planes) to assess myocardial and pulmonary capillary permeability (transfer constant (K^{trans})), pulmonary extracellular volume fraction (Ve) and blood flow (F). GBCA (gadoterate meglumine (Dotarem), Guerbet, France; 3ml/s) administration was divided into three doses in order to avoid saturation of the peak of the arterial input function (AIF), which occurs as a result of the non-linear relationship between contrast agent concentration and signal intensity, and T2 shortening effects, at higher contrast agent concentrations (Roberts TP. Physiologic measurements by contrast-enhanced MR imaging: expectations and limitations. J Magn Reson Imaging. 1997;7(1):82-90.). The initial low dose (0.005 mmol/kg) bolus was used to calculate the AIF first pass peak, followed by 2 minutes of dynamic acquisitions with a temporal resolution equal to 50% of heart rate. Then a higher GBCA dose (0.05 mmol/kg) was administered and followed by 6 minutes of dynamic acquisitions. The higher dose provided higher contrast to noise ratio for the tail of the AIF and for the myocardial and pulmonary tissue curves. The low dose curve was multiplied by 10 and combined with the high dose curve to produce the final AIFs for the kinetic analysis. The third contrast agent dose (0.1 mmol/kg) was administered after the dynamic acquisition to allow LGE imaging and post-contrast T1 mapping.

2. MRI Analysis

MRI analysis was previously described in detail (Lagan J, Naish JH, Bradley J, Fortune C, Palmer C, Clark D, et al. Cardiac involvement in cystic fibrosis evaluated using cardiopulmonary magnetic resonance. Int J

Cardiovasc Imaging. 2022:1-11 [Online ahead of print], Lagan J, Naish JH, Fortune C, Bradley J, Clark D, Niven R, et al. Myocardial involvement in eosinophilic granulomatosis with polyangiitis evaluated with cardiopulmonary magnetic resonance. Int J Cardiovasc Imaging. 2021;37(4):1371-81.). It included the following:

Myocardial function: Cardiac volumetric analysis was performed using Circle CVI42 (Circle Cardiovascular Imaging, Canada) according to current guidelines (Schulz-Menger J, Bluemke DA, Bremerich J, Flamm SD, Fogel MA, Friedrich MG, et al. Standardized image interpretation and post processing in cardiovascular magnetic resonance: Society for Cardiovascular Magnetic Resonance (SCMR) board of trustees task force on standardized post processing. J Cardiovasc Magn Reson. 2013;15(1):35.).

Myocardial and lung tissue characterisation: T1 and T2 maps were generated in Siemens Argus (Siemens Medical Imaging) and transferred into Horos (Horos2K v2.2.0 The Horos Project) where epicardial, endocardial and blood pool regions of interest (ROI) were drawn. Partial volume effects of blood were minimised in myocardial ROIs by using the middle third of myocardium. Lung borders were contoured in Horos, excluding the cardiac outline, to define whole-lung ROIs. Individual lobes were identified as previously described (Donnola SB, Dasenbrook EC, Weaver D, Lu L, Gupta K, Prabhakaran A, et al. Preliminary comparison of normalized T1 and non-contrast perfusion MRI assessments of regional lung disease in cystic fibrosis patients. J Cyst Fibros. 2017;16(2):283-90.). Myocardial extracellular volume (ECV) was calculated using same-day haematocrit as we have described previously (Miller CA, Naish JH, Bishop P, Coutts G, Clark D, Zhao S, et al. Comprehensive validation of cardiovascular magnetic resonance techniques for the assessment of myocardial extracellular volume. Circ Cardiovasc Imaging. 2013;6(3):373-83.). LV extracellular matrix mass (g) was calculated by multiplying LV mass (g) by ECV (%). LV cellular mass (g) was calculated by multiplying LV mass by (100% – ECV).

Myocardial and lung blood flow characteristics: DCE imaging was analysed using custom written Matlab code (v9.0, The MathWorks, USA). Cardiac motion correction was achieved using an intensity-based rigid registration algorithm. Lung registration was performed using Advanced Normalisation Tools (ANTs) symmetric normalization non-linear registration algorithm, employing cross-correlation as the similarity measure (Avants BB, Epstein CL, Grossman M, Gee JC. Symmetric diffeomorphic image registration with cross-correlation: evaluating automated labeling of elderly and neurodegenerative brain. Med Image Anal. 2008;12(1):26-41.). Epicardial, endocardial and lung ROIs were drawn as described above. Arterial input functions (AIF) were derived from the right ventricular (RV) blood pool for the lungs and the LV blood pool for the myocardium. Care was taken to avoid any trabeculations or papillary muscles. Contrast agent kinetics were modelled using the extended version of the Kety model on a voxel-by-voxel basis within registered ROIs to calculate K^{trans} and Ve (Naish JH, Kershaw LE, Buckley DL, Jackson A, Waterton JC, Parker GJM. Modeling of contrast agent kinetics in the lung using T1-weighted dynamic contrast-enhanced MRI. Magn Reson Med. 2009;61(6):1507-14.). Pulmonary blood flow (F) was calculated by deconvolution of the first-pass dynamic data as described previously (Naish JH, Kershaw LE, Buckley DL, Jackson A, Waterton JC, Parker GJM. Modeling of contrast agent kinetics in the lung using T1-weighted dynamic contrast-enhanced MRI. Magn Reson Med. 2009;61(6):1507-14., Hueper K, Parikh MA, Prince MR, Schoenfeld C, Liu C, Bluemke DA, et al. Quantitative and semiquantitative measures of regional pulmonary microvascular perfusion by magnetic resonance imaging and their relationships to global lung perfusion and lung diffusing capacity: the multiethnic study of atherosclerosis chronic obstructive pulmonary disease study. Invest Radiol. 2013;48(4):223-30.).
